# S13 Optimising the inclusivity of mass participation physical activity initiatives: lessons from 20 years of parkrun

**DOI:** 10.1093/eurpub/ckae114.254

**Published:** 2024-09-26

**Authors:** Steve Haake

**Affiliations:** Sheffield Hallam University, Sheffield, England

## Abstract

Over 20 years, parkrun has evolved from a small time trial in London, to a global physical activity and volunteering initiative with over 100 million instances of participation at a Saturday 5km parkrun or Sunday 2km junior parkrun. Every week throughout the year, parkrun takes place at more than 1,800 locations across 22 countries. parkrun welcomes people of all ages, backgrounds and abilities, with the average finish time getting progressively slower. Volunteering is considered an equal form of participation and walking is encouraged.

Yet certain groups face unique and complex barriers to mass-participation physical activity initiatives and are often underrepresented. As parkrun grows, inclusive strategies are needed to optimise inclusivity.

This symposium will explore the experience of parkrun for three priority, often underrepresented, groups; 1) people with common mental health conditions; 2) those at risk of poor mental and social wellbeing; and 3) those with vision impairment. The influence of the ‘parkwalk’ initiative will be discussed as a possible solution for optimising the inclusivity of parkrun. Speakers will reflect on how the learnings from parkrun research can be applied to other health-enhancing physical activity initiatives.

**Presentations:**

1. The experience of parkrun by those with mental health conditions: results from the parkrun UK Health and Wellbeing Survey 2018

2. “I enjoyed the camaraderie, the atmosphere and the inclusiveness of parkrun”: A qualitative study to explore mental wellbeing in middle-aged men at parkrun Ireland

3. “I’m just one of the runners, one of the walkers”. Exploring the experiences of participants who are vision impaired at parkrun in Ireland

4. Using the socio-ecological model of physical activity to test the efficacy of a social intervention at an active leisure event: the parkwalker initiative

**Interactive Q&A:**

Prof Steve Haake (Chair of the parkrun Research Board) will lead a Q&A session with the audience and will start by asking the speakers how inclusivity could be improved. The Chair will invite questions from the audience and curate the discussion to ensure a lively debate about ‘the lessons from parkrun’.

The symposium slides and a summary of discussion topics will be made available online after the conference.

